# Early Life Cognitive Abilities and Body Weight: Cross-Sectional Study of the Association of Inhibitory Control, Cognitive Flexibility, and Sustained Attention with BMI Percentiles in Primary School Children

**DOI:** 10.1155/2015/534651

**Published:** 2015-03-19

**Authors:** Tamara Wirt, Anja Schreiber, Dorothea Kesztyüs, Jürgen M. Steinacker

**Affiliations:** Division of Sports and Rehabilitation Medicine, Department of Internal Medicine II, Ulm University Medical Centre, Frauensteige 6, 89075 Ulm, Germany

## Abstract

The objective of this study was to investigate the association of different cognitive abilities with children's body weight adjusted for further weight influencing sociodemographic, family, and lifestyle factors. Cross-sectional data of 498 primary school children (7.0 ± 0.6 years; 49.8% boys) participating in a health promotion programme in southwest Germany were used. Children performed a computer-based test battery (KiTAP) including an inhibitory control task (Go-Nogo paradigm), a cognitive flexibility task, and a sustained attention task. Height and weight were measured in a standardized manner and converted to BMI percentiles based on national standards. Sociodemographic features (migration background and parental education), family characteristics (parental body weight), and children's lifestyle (TV consumption, physical activity, consumption of sugar-sweetened beverages and breakfast habits) were assessed via parental questionnaire. A hierarchical regression analysis revealed inhibitory control and cognitive flexibility to be significant cognitive predictors for children's body weight. There was no association concerning sustained attention. The findings suggest that especially cognitive abilities known as executive functions (inhibitory control and cognitive flexibility) are associated with children's body weight. Future longitudinal and intervention studies are necessary to investigate the directionality of the association and the potential of integrating cognitive training in obesity prevention strategies. This trial is registered with ClinicalTrials.gov DRKS00000494.

## 1. Introduction

The dramatically increased prevalence of childhood obesity in industrialised nations has been declared as a major topic of public health in the recent decade [1, 2]. In Germany, 14.8% of children aged 2 to 17 years are overweight or obese [3]. An increase of overweight and obesity is particularly evident at the age of school entry, between 5 and 8 years [3, 4]. Given the significant adverse biopsychosocial consequences of paediatric overweight, its relatively stable course, and the enormous economic costs to the healthcare system, effective prevention strategies are needed [1, 5–8]. It is therefore important to better understand correlates of paediatric overweight and to identify risk factors. Besides a genetic predisposition, increased body weight is influenced by certain behavioural and lifestyle factors such as unfavourable dietary habits, for example, high consumption of sugar-sweetened beverages or skipping breakfast, low levels of physical activity, and preference of sedentary activities, for example, screen media use [9–12]. Furthermore, cultural and family characteristics such as migration background, low socioeconomic position, low parental education, and parental obesity are associated with childhood overweight [3, 11, 13, 14].

Additionally, there is a growing body of evidence suggesting an association between increased body weight and altered cognitive functioning in children. Overweight children show, for example, worse school performance compared to their normal-weight counterparts [15–17]. Moreover, a negative association between obesity and executive functions has been reported [18]. Executive functions are defined as higher-order control processes of the cognitive system that are related to self-regulation and underlie goal-directed and adaptive behaviour [19, 20]. These processes have already been positively related to social and emotional skills, school success, mental and physical health, and social status in adulthood [20, 21]. Different components of executive functions such as inhibitory control (the ability to withhold inappropriate actions) or cognitive flexibility (the ability to adjust to changed circumstances or demands) are usually distinguished [20]. Regarding the association between obesity and executive functions studies have mainly focussed on inhibitory control which is significantly related to body mass index in children and adolescents [18, 22, 23]. Concerning other executive functions (e.g., cognitive flexibility) or further cognitive domains (e.g., attention, memory, and general cognitive function), however, findings are scarce and inconsistent [18, 24, 25].

Assuming an association between children's body weight and cognitive functioning one possible underlying mechanism may be that certain cognitive abilities play a role in learning, adopting, and maintaining health behaviour [26, 27]. As previously mentioned executive functions and most of all the inhibitory components are related to cognitive self-regulation and to disciplined behaviour [20]. In the context of paediatric obesity inhibitory control may be important for young children to regulate their physical activity level and their food intake in terms of appreciating rules from parents or teachers, resisting temptations (e.g., consumption of sweets when not allowed and watching TV or video games when otherwise engaged), controlling distracting thoughts and negative emotional states which may increase appetite, and staying focused on activities such as playing games. Cognitive flexibility may be critical for children when trying out new behaviour and dealing with changes, barriers, or different settings throughout their day and when deliberate attention control (focusing and switching) is necessary. Appreciating healthier food and beverages and active ways of transport when introduced by caregivers, coping with school entry and the related changes, switching between sedentary activities such as homework and active play, and finding ways of being physically active despite bad weather or without any toys may be a few examples. Besides these control functions, further abilities such as sustained attention may play a role in terms of maintaining the focus of attention on specific activities over a certain period of time. Thus, it is important for children not only to cope with immediate distractions, changes, and temptations, but also to stay focused in the long run.

However, the small body of literature regarding childhood and especially early school age can be criticised. Most studies focused only on older children or adolescents. Selectivity, small size of study samples, and the use of self-reporting measures further limit validity of research results. Moreover, researchers addressing paediatric obesity always emphasise the importance of controlling for social factors such as parental income or education [15]. The objective of the present study, therefore, was to investigate the association between different cognitive abilities (inhibitory control, cognitive flexibility, and sustained attention) and body weight in a large nonclinical sample of primary school children. To consider the outlined methodical issues objective standardised tests and assessment methods were used and potentially confounding factors including sociodemographic features, family, and lifestyle were controlled.

## 2. Materials and Methods

### 2.1. Overview

In the context of a large evaluation study of a school-based health promotion programme in southwest Germany (the Baden-Württemberg Study) cognitive, anthropometric, sociodemographic, and behavioural data of primary school children were collected. The Baden-Württemberg Study was approved by the institutional ethics committee and is registered at the German Clinical Trials Register (DRKS00000494). Teachers of school classes in the federal state of Baden-Württemberg volunteered to participate in the study and written informed consent was obtained from parents prior to data collection. The Baden-Württemberg Study is a longitudinal study and is designed as a randomised controlled trial. A detailed description of the study has been published by Dreyhaupt et al. [28]. For the present analysis only baseline data of the control and intervention group were used. Baseline assessment took place in autumn 2010 (within a 3-month period from the end of summer vacation in September to the beginning of autumn vacation in November). During this time a research group from the University of Ulm visited the participating school classes (one or two classes each day). Thus, all measurements were performed on-site at school during one school day. On the day of a school visit, children were assigned to small groups based on gender and class to perform the different measurements (e.g., cognitive testing and anthropometric measurement). To obtain information about sociodemographic and lifestyle characteristics a parental questionnaire was issued directly after the measuring period (November 2010) and returned within six weeks.

### 2.2. Participants

The total sample of the Baden-Württemberg Study consisted of *n* = 1944 children from ethnically and socioeconomically diverse primary schools in the federal state of Baden-Württemberg, Germany. Primary school classes were recruited using a number of different public relations activities such as written information for schools, education and health authorities, adverts in training catalogues for teachers, informative events, or participation at pedagogic trade shows. For logistical reasons (distances between schools, scope of measurements of the Baden-Württemberg Study, and technical equipment) cognitive testing was only carried out in the southern part of Baden-Württemberg at a convenient distance of the research centre in Ulm. Furthermore, children who were absent on the day of school visit were not retested. Cognitive data collection took place in a subsample of *n* = 513 children. After exclusion of *n* = 15 children due to motor impairment, colour blindness, or lack of compliance the sample for the present analysis amounts to *n* = 498 participants. Children attended either 1st grade (57.0%) or 2nd grade and averaged 7.0 ± 0.6 years of age (range 5–9); 49.8% were boys.

**Figure 1:**
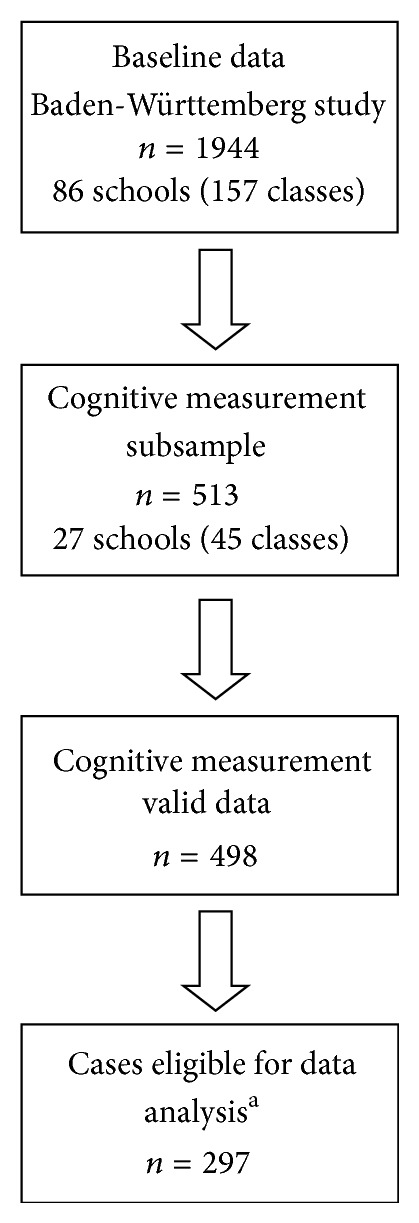
Overview of sample size. ^a^Cases with complete data on cognitive, anthropometric, sociodemographic, family, and lifestyle variables were considered eligible for analysis.

Sample size for each cognitive subtest varies due to further missing or invalid data: *n* = 479 children provided valid data for inhibitory control, *n* = 445 for cognitive flexibility, and *n* = 466 for sustained attention. Reasons for further subtest dropouts were, for instance, time restriction at school and lack of comprehension or compliance or implausible data concerning only one subtest. Anthropometric data was available for *n* = 496, and the parental questionnaire was filled out for *n* = 441 children. Complete data including all cognitive measures, anthropometric measures, and parental questionnaire was available for *n* = 297.  Figure 1 provides an overview of the sample and subsample selection.

### 2.3. Cognitive Measures

Cognitive abilities were assessed using the computer-based test battery of attention for children (KiTAP) [29]. The KiTAP is validated for children aged 6 to 10 years and consists of a broad range of nonverbal subtests measuring different basal as well as higher-order components of the cognitive system (attention and executive functioning). Each component can be assessed separately. To ensure optimal motivation and compliance all subtests are designed in the form of short games with an enchanted castle theme. This allows the KiTAP to be particularly accessible to young children in comparison to other known test batteries based on more abstract stimuli. Furthermore, a computer-based test was preferable to a paper pencil test as preliminary trials demonstrated that children just entering school had difficulties in turning pages and handling a pencil. Due to the child-friendly character, the feasibility in the school and group setting, and the possibility to measure differentially cognitive functioning (including executive control components) the KiTAP constituted a suitable assessment tool for the present study purposes. In terms of validity the test battery has been especially used in neuropsychological and other paediatric researches [30–32] as well as in research with healthy children and in cross-cultural studies [33, 34]. Significant correlations with school outcomes [34], intellectual abilities [32], and behavioural questionnaires [35] could be found. Factorial analysis confirmed the construct validity [29], and group comparisons (e.g., children with versus without attention deficit hyperactivity disorder) demonstrated criterion validity [30]. The reliability of the test battery can be considered as satisfactory [29].

Three subtests of the KiTAP were administered: an inhibitory control task (Go-Nogo paradigm), a cognitive flexibility task, and a sustained attention task. For each task number of errors (incorrect response to a noncritical stimulus), number of omissions (missed response to a critical stimulus), and reaction time (milliseconds in medians) were recorded. For statistical analysis and to overcome the right skewed distributions of errors and omissions total scores were calculated for each subtest based on the key parameters recommended in the test manual.

*(1) Inhibitory Control*. The Go/Nogo task examined the ability to respond as quickly as possible to a certain critical stimulus by pressing a button and to withhold the response when another noncritical stimulus emerged. The task lasted 3 minutes. Key parameters were errors and reaction time. Errors could range from 0 to 20 and reaction time from 0 ms to 2700 ms (maximum time interval between two stimuli). The total score was calculated as follows:

(1) Total  score=errors  standard  scores −reaction  time  standard  scores.

To improve interpretability the score was reversed with a positive total score indicating an overly high inhibitory control (low number of errors and slow reflexive reactions) and a negative score indicating low inhibitory control (high number of errors and fast impulsive reactions). A score around 0 represented an average inhibitory ability.

*(2) Cognitive Flexibility*. The task examined the ability to deliberately control the attention focus and to adapt responses to changing conditions as quickly as possible. Children had to consider different features simultaneously (colour and location of the stimulus), to switch their attention continuously between these features, and to react appropriately according to the target feature in each trial. In detail, two stimuli in two different colours were presented simultaneously on the right and the left sides of the screen. Children had to press one out of two buttons (left button for the left side or right button for the right side) depending on the colour of the stimuli in an alternate sequence (colour A, colour B, colour A, colour B,…). On each trial, the stimulus with the target colour could be presented on the same side of the screen as before or on the other side; thus, children had to change their response behaviour or not. Duration of the whole task varied depending on reaction times (approximately 3 minutes). Key parameters were errors and reaction time. Errors could range from 0 to 50 and reaction time from 0 ms to 60000 ms (maximum time interval between two stimuli if no reaction occurred). Contrary to the other subtests, a total score was automatically computed by the KiTAP based on standardised number of errors and reaction time [29]. A positive score represented overly high flexibility (low number of errors and fast reactions) and a negative score low flexibility (high number of errors and slow reactions). A score around 0 represented average cognitive flexibility.

*(3) Sustained Attention*. The task examined the ability to maintain attention over an extended period of time (10 minutes). During this time children had to compare subsequent stimuli in terms of a specific feature (colour) and to determine whether two stimuli were matching. Key parameters were errors (two stimuli incorrectly indicated as matching) and omissions (two stimuli incorrectly indicated as nonmatching). Errors could range from 0 to 250 and omissions from 0 to 50. The total score represented the number of correct responses and was calculated as follows:

(2) Total  score =total  number  of  stimuli  −number  of  errors+number  of  omissions.

To consider the different number of errors and omissions possible (250 versus 50) the number of errors was relativised (divided by 5):

(3) Total  score =100−number  of  errors5+number  of  omissions.

Thus, the total score ranged from 0 to 100 with a high total score indicating high sustained attention and a low score indicating low sustained attention. A score around 0 represented no sustained attention at all.

*Procedure*. On the day of a school visit the cognitive tests were administered during the first school hours. Cognitive testing took place in one or two separate quiet classrooms and was carried out by trained examiners using laptops (screen size: 15 inches). As previously mentioned children performed the tests in small groups (up to 8 children). Per group 4 examiners supported and supervised the children (with a maximum of 2 children per examiner). While one testing session took place, which lasted in total 30 minutes, the other groups were assigned either to anthropometric measurement or to other parts of the Baden-Württemberg Study. The subtests of the KiTAP were administered in a fixed order and instructions were given in a standardised manner. Comprehension and willingness of the children were assured by short preceding practice trials according to the test manual. These practice trials could be repeated if necessary—especially the cognitive flexibility task required several preceding trials. The main testing started when it was clear that each child of the group understood the instructions. When the examiner was sure that a child was not able to perform a task, lack of comprehension was documented. The main testing was administered once. Further irregular and disruptive behaviour was documented and later considered during data preparation. Children who were absent on the day of testing were excluded from the analysis as there was no repetition of the testing at a later point in time.

### 2.4. Anthropometric Measures

Anthropometric measurement took place in a separate room provided by the teacher. Gender segregation of the groups was considered. Body height and weight of the children were taken by trained staff according to the guidelines of the International Society for the Advancement of Kinanthropometry (ISAK) [36]. Height was measured using a portable stadiometer (Seca model 217, Seca, Germany), without wearing shoes, with an accuracy of 0.1 cm. Weight was measured using a calibrated electronic scale (Seca model 862, Seca, Germany), wearing underwear, with an accuracy of 0.05 kg. Children's body mass index (BMI) was calculated as weight divided by height squared (kg/m²) and converted to BMI percentiles using national age- and sex-specific reference data [37]. To allow international comparisons, weight status was also calculated according to international reference data [38].

### 2.5. Parental Questionnaire

Sociodemographic data, body weight of parents, and different lifestyle factors of children were assessed via parental questionnaire. Parent education was assigned to the respective level according to the CASMIN classification [39]. The CASMIN (Comparative Analysis of Social Mobility in Industrial Nations) is the most widely used international instrument to classify education considering length, quality, and value of general education as well as vocationally oriented schooling or training. The classification distinguishes primary, secondary, and tertiary education levels. In the present study parent education was determined as the highest level of two parents or the level of a single parent who cares for the child. Due to the small number of cases with primary education level (1.0%) parent education was dichotomised with primary and secondary education levels in one group and tertiary education level in another. Migration background was defined as at least one parent born abroad or at least one parent mainly having spoken a foreign language with the child during its first years of life. Self-reported parental height and weight were used to calculate BMI of mothers and fathers (kg/m²). Concerning children's lifestyle, TV consumption, physical activity, consumption of sugar-sweetened beverages, and breakfast habits were assessed. The mean time spent watching television per day was rated on a 7-point Likert scale (“never” to “more than 4 hours”). As the American Academy of Paediatrics [40] recommends less than 1-2 hours of total screen time per day, TV consumption was dichotomised using a cut-off point at 1 hour. Further, parents were asked on how many days per week their child was engaging in at least 60 minutes of moderate to vigorous physical activity (range 0 to 7 days) and how often their child was consuming sugar-sweetened beverages (6-point Likert scale: “never” to “more than once per day”). The frequency of having breakfast prior to going to school was rated on a 4-point Likert scale and, for statistical analysis, dichotomised as “never”/“rarely” versus “often”/“always.”

### 2.6. Statistical Methods

To determine the additional predictive value of each of the three cognitive variables on children's body weight (BMI percentiles) hierarchical multiple linear regression analysis was performed. First, a basic model (model 1) was established which included parent education, migration background, BMI of mother and father, children's TV consumption, physical activity, consumption of sugar-sweetened beverages, and breakfast habits. In the next steps inhibitory control, cognitive flexibility, and sustained attention were added successively as predictors (models 2 to 4). Statistical analysis was carried out using SPSS 19 and statistical significance was set at *α* = 0.05. As missing data may have had an impact on the results, differences between the samples and subsamples (the Baden-Württemberg Study sample, *n* = 1944, the cognitive subsample, *n* = 498, and the final sample with complete and valid data, *n* = 297) were analysed using *t*-test for continuous data and Fisher's exact test for categorical data.

## 3. Results

### 3.1. Descriptive Characteristics

Sociodemographic, lifestyle, and weight group characteristics of the different samples are shown in Table 1. In the cognitive subsample (*n* = 498) average BMI percentile of children was 48.21 ± 26.92, and 8.4% were classified as overweight or obese and 7.3% were classified as underweight according to national standards [37]. The prevalence for overweight and for underweight was slightly higher according to international cut-off points [38] (Table 1). Parental BMI averaged 23.86 ± 4.47 (mothers) and 27.90 ± 3.93 (fathers), respectively. Means and standard deviations for all cognitive subtests (total scores, number of errors, number of omissions, and reaction time) are illustrated in Table 2.

### 3.2. Prediction of Body Weight

To determine whether different cognitive abilities are associated with children's body weight hierarchical regression analysis was conducted. Results are presented in Table 3. First, model 1 revealed migration background, body weight of mother, and body weight of father as significant predictors of children's body weight. No relationship between parental education or the different lifestyle factors and children's body weight was found. As it is shown in models 2 to 4 inhibitory control and cognitive flexibility were significant cognitive predictors over and above all other variables whereas sustained attention did not significantly contribute to the prediction. Inhibitory control and cognitive flexibility together explained an additional amount of 4.5% of variance in the criterion.

### 3.3. Missing Data

Children of the cognitive subsample (*n* = 498) differed from children of the total study population in terms of migration background and father's BMI. A significantly higher percentage of migration background (*P* = 0.022) and a significantly lower father's BMI (*P* = 0.001) were found in children who performed the KiTAP compared to those who did not. There were no significant differences concerning age, sex, BMI percentiles, weight group, TV consumption, physical activity, consumption of sugar-sweetened beverages, breakfast habits, parental education, and mother's BMI. Children of the final subsample with complete data (*n* = 297) differed significantly from children of the total study population in terms of BMI percentiles, parental education, consumption of sugar-sweetened beverages, and father's BMI. Lower BMI percentiles (*P* = 0.008), a higher percentage of tertiary parental education level (*P* = 0.009), a lower percentage of soft drink consumption (*P* = 0.031), and a lower father's BMI (*P* = 0.000) were found in children with complete data compared to those without. There were no significant differences concerning any other variable. Although the percentage of migration background was increased in the cognitive subsample, more children with migration background dropped out in the further data process. Thus, the final subsample did not differ anymore from the total study sample in this respect.

## 4. Discussion

The present study examined the association between different cognitive abilities and body weight in primary school children. The findings suggest that especially cognitive abilities known as executive functions such as inhibitory control and cognitive flexibility are associated with children's body weight. In the past decade particularly the influence of inhibitory control was investigated in children and adolescents using a variety of assessment tools [18]. Methods ranged from behaviour questionnaires, ratings, and self-reports to different tasks and computerised tests (e.g., Stroop test, Go-Nogo task, and delay-of-gratification task). In line with the results reported here all studies showed a significant relationship between body weight and inhibitory control in that a higher body weight was associated with poorer inhibition performance. Additionally, a few longitudinal studies indicated that inhibitory control at a younger age can predict children's BMI at an older age [18, 22, 41]. Group analyses revealed less inhibitory control in overweight adolescents compared to their normal weight peers [18, 42]. Pauli-Pott et al. [23] further pointed out a significant interaction with age and assumed that there might be an especially important developmental period at early school age when inhibitory control is particularly important for self-regulation.

Few studies can be found examining the association between cognitive flexibility and body weight. Cserjési et al. [24], for example, found a significant negative correlation in adolescent boys, and obese boys significantly performed worse than their healthy weight counterparts. Verdejo-García et al. [42] used a whole battery of executive functioning tests including response inhibition and flexibility. Similarly, the authors reported significant group differences in the flexibility task and a significant relationship between BMI and flexibility. These findings are supported by further studies focusing all mainly on adolescents [43, 44], whereas Gunstad et al. [25, 45] demonstrated a link between cognitive flexibility (switching-of-attention task) and body weight only in adults but neither in children nor in adolescents. The results reported here conform to most of the existing research literatures even though these studies have been conducted mainly in older children. Thus, besides inhibitory control another executive functions domain seems to be associated to body weight and weight gain and, according to the present finding, this seems to be true in younger children, too.

On the other hand, the third cognitive domain, sustained attention, was not related to BMI percentiles in the current investigation. Previous findings concerning cognitive abilities other than executive functions are inconsistent. The literature review of Reinert et al. [18] reports six studies focusing on the association between obesity and general cognitive function with half of them demonstrating no relationship. Graziano et al. [22] considered sustained attention besides inhibitory control as part of cognitive self-regulation. Body weight of their preschool children, however, was only associated with the inhibitory performance but not with the attention performance. On the contrary, Cserjési et al. [24] showed the same result as for flexibility in their adolescent sample: a significant correlation of performance in the D2 sustained attention test with BMI and a significant group difference to the disadvantage of the obese. The existing inconsistencies in research literature might be due to the different age groups and to the use of different concepts and methods of the studied cognitive abilities. Hence, standardisation concerning the understanding and measurement of certain cognitions should be targeted and changes in outcomes according to stages of development should be taken into account when addressing this issue.

Besides the cognitive variables, parental body weight, BMI of mothers as well as BMI of fathers, was significantly associated with children's body weight. This finding is not surprising as it is consistent with the literature [11, 12, 14] and may be explained by genetic mechanisms as well as the shared environment. Family characteristics such as the knowledge of risk factors of overweight, eating habits, and food preferences but also physical activity patterns [46] may influence children's health behaviour and body weight. Migration background was revealed to be significantly associated with body weight as well. This finding is in line with previous national investigations [3, 11, 13]. The prevalence of overweight and obesity is found to be higher in children with migration background and the odds of overweight increased. Cultural attitudes and traditions concerning body weight and weight related behaviours (physical activity, TV consumption, and dietary habits), social integration (e.g., influencing recreational activities), and the knowledge of risk factors hampered by language barriers may explain this relationship.

Executive functions are seen to be crucial for self-regulatory behaviour [47]. They have already been related to health behaviour such as physical activity, snack food consumption, and fruit/vegetable intake in fourth graders [26]. Thus, the association with children's body weight may be mediated through more physical activity and healthy diet and less sedentary behaviour. As children just starting school are still more dependent on their parents and not completely autonomous in their planning and decision-making executive functions may, however, be crucial to appreciate and maintain new and healthy behaviour introduced by their caregivers, to control their thoughts, their behavioural impulses, and their feelings. Assuming this directionality, potential implications would be to integrate the promotion of executive functions in early obesity prevention efforts. Riggs et al. [48] suggested developing specific programme contents tailored to different obesity-risk profiles depending on certain behaviour patterns, weight consciousness (especially as children get older), and deficits in executive functions. Beyond the overweight and obesity issue, it has been shown that executive functions play an important role for success and health throughout the whole life. They are crucial for the social and emotional development, school readiness, and further academic and job success, as well as wealth and mental and physical health even in the long term [20, 21, 49, 50]. Regulating emotions in social conflicts, staying in control of oneself, adapting to rules when necessary, adopting effective problem-solving, and learning strategies are just a few examples when executive control is required. On the contrary, deficits are linked to social and health problems such as attention deficit hyperactivity disorder, obsessive compulsive disorder, depression, early school leaving early pregnancy, addiction, and criminality [20, 21]. Hence, strategies focusing on the improvement of these abilities would probably lead to positive effects in more than one health and life domain and even on a more public level (e.g., public safety and economic costs). In return, learning to cope with the different challenges in life successfully and to reduce emotional and social stress means reducing psychological risk factors for excessive weight gain again and starting a virtuous circle. There have already been national and international efforts aiming at an early improvement of executive functions in general [20, 50, 51]. These include school-based programmes and the integration of the promotion of these abilities in the official curriculum of primary schools in Germany. Thus, cognitive training in general and the integration of cognitive improvement in obesity interventions may be helpful ways to improve future generation's health and overall quality of life.

### 4.1. Strengths and Limitations

Results, however, should be interpreted in light of study limitations. First, the cross-sectional study design precludes any causal interpretation of the findings. Therefore, directionality of the association between cognitive functions and body weight still remains unclear: on the one hand, cognitive functions such as inhibitory control or cognitive flexibility may influence health behaviour and consequently weight development. On the other hand, body weight and variations in food intake, physical activity, and sedentary activities, for example, may also affect cognitive performance and brain development or the relationship may be bidirectional as well. Further studies are needed to clarify causality and underlying mechanisms in order to derive any implications.

Secondly, there are some limitations concerning missing data and selection bias in the present study. Due to the subsample and the missing or invalid data in the cognitive subtests and the parent questionnaire the number of subjects decreased from 1944 (in the Baden-Württemberg Study sample) to 297 in the final regression analysis. Missing data may have led to a form of selection bias. The cognitive subsample included, for example, more children with migration background. However, more children with migration background and with lower parental education level dropped out in the further data processing maybe partly due to comprehension difficulties. Furthermore, more children with higher BMI percentiles, higher consumption of sugar-sweetened beverages, and higher father's BMI were among those with missing data. Thus, children who entered the final analysis showed a more favourable profile in critical variables. Although migration background is still representative for school children in Germany, the final sample consisted of more children with higher parental education indicating a higher social status and lower body weight than usually found in the population (as reference, official statistics concerning German school children report 32.1% migration background, 23.9% tertiary parental education level, and 13.3% overweight or obesity [3, 52, 53]). On the contrary, the reduced sample size and statistical power may have led to an underestimation of significances. The inclusion of these missing cases could have potentially strengthened the final results. Furthermore, this may in part explain why no significant association between parental education and children's body weight was found.

Finally, underreporting in terms of recall bias or social desirability regarding children's lifestyle which was assessed via parental questionnaire should be taken into account and might explain the missing significant association of these variables with body weight. The objective standardised and direct measurements of cognitive and anthropometric data, on the other hand, as well as the large sample size constitute a strength of the study. Further, the focused age group is highly relevant as excessive weight gain is particularly pronounced at the age of school entry and important cognitive developments especially in executive functions relevant for a wide variety of behaviour and health outcomes take place.

## 5. Conclusions

In summary, cognitive abilities were significantly related to body weight of primary school children controlling for further weight influencing sociodemographic and lifestyle factors. This relationship concerns inhibitory control and cognitive flexibility, both processes considered as executive functions. As executive functions are crucial for self-regulation and disciplined behaviour including health behaviour, the finding indicates that promoting executive functions may assist in developing a healthy body weight and avoiding excessive weight gain in addition to already existing obesity prevention efforts. However, further research is necessary first, in particular longitudinal and intervention studies, to confirm the present findings, to determine the directionality of the association, and to investigate the impact of cognitive training on weight related outcomes.

## Figures and Tables

**Table 1:** Descriptive characteristics of the Baden-Württemberg Study sample and the KiTAP subsamples.

	Baden-Württemberg Study sample (*n* = 1944)	Missing values	Cognitive subsample (*n* = 498)	Missing values	Final subsample^a^ (*n* = 297)
Child characteristics					
Age, m (sd)	7.1 (0.6)	0	7.0 (0.6)	0	7.1 (0.6)
Female, *n* (%)	949 (48.8)	0	250 (50.2)	0	141 (47.5)
BMI percentiles, m (sd)	49.0 (27.9)	51	48.2 (26.9)	2	45.2 (26.3)
Weight group, national reference data, *n* (%) [37]					
Underweight (<10 BMI percentile) Overweight (>90 and ≤97 BMI percentile) Obese (>97 BMI percentile)	148 (7.8) 108 (5.7) 82 (4.3)	51	36 (7.3) 25 (5.0) 17 (3.4)	2	25 (8.4) 14 (4.7) 6 (2.0)
Weight group, international reference data, *n* (%) [38]					
Underweight Overweight Obese	78 (4.0) 190 (10.0) 74 (3.9)		50 (10.0) 46 (9.2) 15 (3.0)		35 (11.8) 22 (7.4) 4 (1.3)
TV consumption > 60 minutes/day, *n* (%)	242 (14.3)	254	65 (14.9)	61	37 (12.5)
Days/week with at least 60 minutes MVPA, m (sd)	2.7 (1.7)	321	2.8 (1.7)	81	2.8 (1.7)
SSB consumption > once/week, *n* (%)	416 (24.4)	242	95 (21.6)	58	58 (19.5)
Never/rarely having breakfast, *n* (%)	223 (13.0)	237	60 (13.7)	58	35 (11.8)
Parental characteristics					
Tertiary parent education level, *n* (%)	522 (32.2)	324	148 (35.4)	80	115 (38.7)
Migration background, *n* (%)	525 (31.9)	298	156 (36.4)	70	97 (32.7)
Mother's BMI	24.1 (4.5)	361	23.9 (4.5)	89	24.1 (4.8)
Father's BMI	28.5 (4.1)	481	27.9 (3.9)	121	27.8 (4.0)

*Note.* ^a^Cases with complete data on cognitive, anthropometric, sociodemographic, family, and lifestyle variables. MVPA = moderate to vigorous physical activity. SSB = sugar-sweetened beverages.

**Table 2:** Mean, standard deviation, and range for cognitive test scores.

	M	SD	Minimum	Maximum	*n*
Inhibitory control					
Total score	−0.01	1.68	−5.48	4.51	479
Number of errors	5.28	3.26	0	15	
Reaction time (ms)	511.14	76.34	298.00	778.00	
Cognitive flexibility					
Total score	−0.60	9.56	−30.40	22.62	445
Number of errors	6.42	3.73	0	16	
Reaction time (ms)	1261.66	305.14	445.00	2290.00	
Sustained attention					
Total score	82.74	8.86	60.60	100.00	466
Number of errors	16.26	16.69	0	72	
Number of omissions	14.00	7.87	0	37	

*Note.* ms = millisecond.

**Table 3:** Hierarchical multiple regression model predicting children's body weight from parental, behavioural, and cognitive variables.

Predictors	BMI percentiles				
	Model 1	Model 2	Model 3	Model 4	
	*B *	*B *	*B *	*B *	95 % CI
Parent education	−4.61	−4.00	−3.91	−4.33	[−10.44, 1.77]
Migration background	7.32^*^	6.68^*^	6.87^*^	6.61^*^	[0.26, 12.97]
BMI of mother	1.22^***^	1.13^***^	1.18^***^	1.20^***^	[0.59, 1.81]
BMI of father	1.15^**^	1.20^**^	1.13^**^	1.11^**^	[0.37, 1.86]
TV consumption	−0.58	−1.99	−2.52	−2.53	[−11.70, 6.64]
Physical activity SSB consumption	1.4 −2.31	1.18 −2.37	0.99 −2.39	0.94 −2.44	[−0.78, 2.65] [−6.07, 1.19]
Breakfast habits	4.69	5.05	6.67	6.53	[−2.50, 15.55]
Inhibitory control		−1.98^*^	−1.94^*^	−1.94^*^	[−3.65, −0.23]
Cognitive flexibility			−0.46^**^	−0.50^**^	[−0.79, −0.20]
Sustained attention				0.15	[−0.18, −0.48]
*R* ^2^	0.14	0.16	0.19	0.19	
*F*	5.78^***^	5.77^***^	6.37^***^	5.86^***^	
Δ*R* ^2^		0.02	0.03	0.00	
Δ*F* ^2^		5.01^*^	10.05^**^	0.77	

*Note*. *N* = 297. CI = confidence interval. SSB = sugar-sweetened beverages. ^*^ *P* < 0.05; ^**^ *P* < 0.01; ^***^ *P* < 0.001.
